# Quantifying the Characteristics of Diabetic Retinopathy in Macular Optical Coherence Tomography Angiography Images: A Few-Shot Learning and Explainable Artificial Intelligence Approach

**DOI:** 10.7759/cureus.76746

**Published:** 2025-01-01

**Authors:** Ali Akbar Movassagh, Mahdie Jajroudi, Amir Homayoun Jafari, Elias Khalili Pour, Hossein Farrokhpour, Hooshang Faghihi, Hamid Riazi, Hossein ArabAlibeik

**Affiliations:** 1 Department of Medical Physics and Biomedical Engineering, School of Medicine, Tehran University of Medical Sciences, Tehran, IRN; 2 Medical Informatics, Mashhad University of Medical Sciences, Mashhad, IRN; 3 Eye Research Center, Farabi Eye Hospital, Tehran University of Medical Sciences, Tehran, IRN

**Keywords:** diabetic retinopathy, explainable ai, few-shot learning, optical coherence tomography angiography, self-attention

## Abstract

Background: Early detection and accurate staging of diabetic retinopathy (DR) are important to prevent vision loss. Optical coherence tomography angiography (OCTA) images provide detailed insights into the retinal vasculature, revealing intricate changes that occur as DR progresses. However, interpreting these complex images requires significant expertise and is often time-intensive. Deep learning techniques have the potential to automate DR analysis. However, they typically require large datasets for effective training. To address the challenge of limited data in this emerging imaging field, a combined approach using few-shot learning (FSL) and self-attention mechanisms within explainable AI (XAI) was explored.

Objective: To investigate and evaluate the potential of an FSL-self-attention XAI approach to improve the accuracy of DR staging classification using OCTA images.

Methods: A total of 206 OCTA images, comprising 104 non-proliferative diabetic retinopathy (NPDR) and 102 proliferative diabetic retinopathy (PDR) cases, were analyzed using the FSL method. Three pre-trained networks (ResNet-50, DenseNet-161, and MobileNet-v2) were employed, with the top-performing model subsequently integrated with the Match-Them-Up Network (MTUNet) to provide explainable interpretations using a self-attention mechanism. The performance of the models was evaluated by applying standard metrics, including accuracy, sensitivity, specificity, and area under the receiver operating characteristic curve (AUC-ROC). The performance of the MTUNet model is assessed by calculating pattern-matching scores for PDR and NPDR classes.

Results: The ResNet-50 pre-trained model in FSL demonstrated the best overall performance, achieving an accuracy of 76.17%, a sensitivity of 81.83%, a specificity of 70.5%, and 0.82 AUC in classifying DR stages. MTUNet provided pattern-matching scores of 0.77 and 0.75 for PDR and NPDR classes, respectively.

Conclusions: FSL and self-attention mechanisms in XAI offer promising approaches for accurate DR stage classification, especially in data-limited scenarios. This could potentially facilitate early DR detection and inform clinical decision-making.

## Introduction

This section can include background information such as theories, prior work, and diabetic retinopathy (DR) is a significant global health issue worldwide, affecting approximately one-third of individuals with diabetes [1-3]. Current screening methods, such as direct ophthalmoscopy, slit-lamp biomicroscopy, and fundus photography, often rely heavily on the expertise of ophthalmologists to interpret complex retinal changes, which is time-consuming and resource-intensive [4,5]. In general, patients with diabetes are commonly categorized into three groups: those without DR, those with non-proliferative DR (NPDR), and those with proliferative DR (PDR) [6].

Optical coherence tomography angiography (OCTA) is a powerful imaging technique that provides a clear visualization of retinal blood flow. By detecting subtle changes in the retinal microvasculature, OCTA can identify early signs of DR, such as microaneurysms and capillary dropout, even before symptoms appear. As DR progresses, OCTA can identify more severe complications, including neovascularization in PDR and intraretinal microvascular abnormalities (IRMAs) in NPDR [6-9].

There is a growing interest in using machine learning (ML) and deep learning (DL) techniques to assist in analyzing OCTA images for DR diagnosis and staging. ML techniques involve the extraction of quantitative features such as blood vessel density (BVD), blood vessel caliber (BVC), blood vessel tortuosity (BVT), vessel perimeter index (VPI), vessel complexity index (VCI), foveal avascular zone (FAZ) area (FAZA), and FAZ contour irregularity (FAZCI) from OCTA images, and then applying models to these features for screening or staging purposes. Various studies have shown that ML can be a valuable tool for detecting diabetes and the early stages of NPDR, as well as the differential between NPDR and PDR and the severity of DR [10-12].

DL-based algorithms enhance the automation of DR screening by analyzing extensive image datasets, such as OCTA images. These algorithms effectively identify at-risk patients, classify DR stages, monitor disease progression, and evaluate treatment efficacy [13-16]. However, deep learning often faces challenges such as limited data and the need for extensive labeling. Few-shot learning (FSL) addresses these challenges by reducing the reliance on large datasets and minimizing the need for manual labeling, making it particularly beneficial for resource-constrained users [17].

Explainable AI (XAI) techniques enhance the interpretability of deep learning models, and their combination with FSL creates a robust framework for accurate, efficient, and interpretable OCTA-based DR classification, even with limited data [18]. The self-attention mechanism in transformer models greatly enhances the explainability of AI by identifying critical regions in input data and illustrating how the model prioritizes these elements during classification. By visualizing attention scores and the distinct roles of various self-attention heads, this mechanism offers insights into model behavior, clarifying how different attention patterns impact performance and decision-making [19,20].

One promising approach to integrating FSL and XAI is the Match-Them-Up Network (MTUNet), which utilizes a pattern extractor to identify distinctive image features and incorporates a pairwise matching mechanism for classification, enabling effective performance with limited labeled data while also employing self-attention mechanisms to prioritize relevant image regions, enhancing interpretability and aligning more closely with the requirements of clinical diagnosis [21].

This study aimed to investigate and evaluate how the FSL framework, combined with XAI through attention mechanisms, can improve the accuracy of DR classification using OCTA images, specifically discriminating between PDR and NPDR stages and highlighting focal points for interpretation.

## Materials and methods

**Figure 1:**
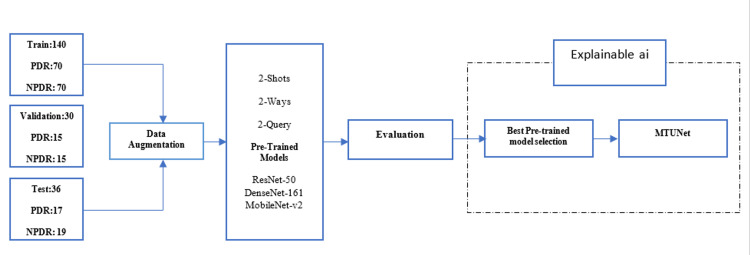
End-to-End Pipeline for Diabetic Retinopathy (DR) Classification Based on Optical Coherence Tomography Angiography (OCTA) imaging Using Few-Shot Learning (FSL) and Explainable AI with Match-Them-Up Network (MTUNet) This diagram provides an overview of the study’s workflow for diabetic retinopathy (DR) classification, starting with the acquisition of Optical Coherence Tomography Angiography (OCTA) images labeled as Proliferative (PDR) or Non-Proliferative (NPDR). Data augmentation techniques are employed to expand the training dataset and improve model generalization. Subsequently, a few-shot learning approach (2-shots, 2-ways, 2-query) is implemented using various pre-trained neural networks (ResNet-50, DenseNet-161, MobileNet-v2) for DR severity classification with limited data. The best-performing model is then integrated into a Match-Them-Up Network (MTUNet) framework to extract relevant features effectively. Finally, Explainable AI (XAI) methods enhance the model's transparency and interpretability by providing clear insights into the classification decisions.

This retrospective study was approved by the IRB of Tehran University of Medical Sciences (IR.TUMS.FARABIH.REC.1403.033) and conducted following the Declaration of Helsinki. The dataset was retrieved from Farabi Eye Hospital, Tehran, from 2022 to 2023. All the participants provided written informed consent. Figure 1 shows a schematic of the steps used in this study.

Image acquisition

OCTA images were acquired using an RTVue XR 100 Avanti device (Version 2017.1.0.151, Optovue, Inc., Fremont, CA, USA). Between 8:00 a.m. and 12:00 a.m., 6 × 6 mm2 superficial, deep, and total (combined) capillary plexuses (SCP, DCP, CCP) [6] were extracted from the en-face macular OCTA images of patients with varying stages of DR. All patients underwent fluorescein angiography (Heidelberg Engineering, Heidelberg, Germany) at baseline for staging of the DR.

Diagnostic criteria of NPDR and PDR, design study, and patients

Two retina experts (H.R.E. and E.K.P.) reviewed fundus exams and fluorescein angiography images to determine retinopathy stages by consensus. Patients with neovascularization at the disc (NVD) or elsewhere (NVE) were categorized as PDR; others were classified as NPDR. A senior physician resolved any disagreements. Finally, the intra-class coefficient (ICC) was calculated. Of the 150 patients examined, 81 were included: 56 NPDR and 25 PDR, with a balanced gender distribution in NPDR (28 each) and 9/16 in PDR. The low quality and artifact of images dropped, and 206 participated in the study. Figure 1 shows that the data will be split into 80% for training and the rest for testing and validation. NPDR was labeled as 0, 1 for PDR.

Pre-processing and augmentation

All images were initially resized to 224 × 224 pixels. Subsequently, an augmentation strategy involving adjustments to the contrast, brightness, and orientation was applied to address the variability in image quality. Specifically, the contrast and brightness levels were randomized for each image to enhance the dataset's diversity and improve the model's robustness in handling varying image conditions.

Algorithms

FSL was applied in two independent phases: first, we used prototyping FSL for classification, and second, MTUNet was applied as XAI.

Prototyping FSL

FSL is an advanced machine learning approach developed to overcome the challenges associated with limited labelled data in training. During FSL, a model is first trained on a small "support set" of labelled samples and then evaluated on a "query set" of unlabeled samples. “n-shot,” “n-query,” and “n-way” are key to this framework, referring to the number of samples per class in the support set, number of samples per class in the query set and the number of classes, respectively.

Various methods have been developed, but prototypical networks, matching networks, relation networks, and the META Learning approach are the most commonly used in FSL. The simplicity, effectiveness, scalability, and interpretability of Prototypical Networks make them well adapted for FSL tasks, even when trained with a minimal amount of labelled data, and therefore highly robust in classifying unseen classes.

The prototypical network employed in this study operated by computing a prototype for each class. It was developed by averaging the feature vectors for each class's support samples. At query time, a sample is classified according to the comparison of its embedding with class prototypes. It is usually performed using distance computation, where the Euclidean distance is most often used. The sample was assigned to the class with the closest prototype [22].

In addition, FSL contains backbone pre-trained models, such as ResNet-50, VGG 16, etc., to address the limited size of the labelled dataset. These models are deep convolutional neural networks that have been pre-trained on large-scale datasets and adapted for FSL, providing rich feature representations that are effectively utilized even with minimal training data.

Pre-training model selection

In selecting and integrating a pre-trained model for few-shot learning, several parameters, including the complexity of the model, task compatibility, feature representations, transferability, and regularization, must be considered to ensure that the model performs well in the new model.

In this study, ResNet 50 [23], DenseNET 16 [24], and Mobile Net [25] were selected as backbone networks, prioritizing a balance between performance and complexity [26]. Figure 1 illustrates the FSL framework employing these backbone networks, with n-way, n-shot, and n-query values set to 2.

Explainable AI: Match-Them-Up Network (MTUNet)

XAI is a field of artificial intelligence focused on making complex machine learning models and their decisions more understandable and interpretable to humans. There are generally two approaches to XAI: post-hoc and intrinsically explainable (ante-hoc). The difference lies in how explainability was introduced into the model. Post-hoc models utilize XAI techniques to evaluate and interpret a model after training, whereas ante-hoc models are naturally interpretable [21].

MTUNet is an ante-hoc explainable few-shot image classification framework that integrates effective few-shot classification with visual representation to ensure improved generalization. Using a pre-trained model, MTUNet first employs a pattern extractor (PE) to convert support images into feature vectors. Prototype computation is then performed for each class by averaging the feature vectors of all the support images belonging to the class, creating representative central reference points for classification. When a query image is provided, the Pairwise Matching (PM) module calculates similarity matching by comparing it to the class prototypes using a distance metric such as the Euclidean distance [21].

**Figure 2:**
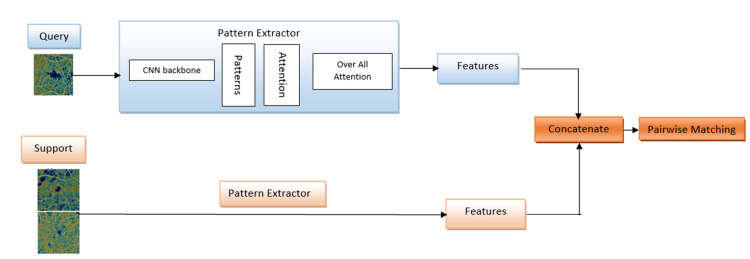
Match-Them-Up Network (MTUNet) architecture for diabetic retinopathy (DR) classification using Few-Shot Learning and attention map for Enhanced Interpretability on Optical Coherence Tomography Angiography (OCTA) images. Schematic diagram of the Match-Them-Up Network (MTUNet) architecture for diabetic retinopathy (DR) classification using Optical Coherence Tomography Angiography (OCTA) images. The model integrates few-shot learning with explainable AI to achieve accurate and insightful results. The process starts with a Query and Support image fed into the Pattern Extractor. This component employs a ResNet backbone and attention mechanisms, including Slot-based Cross-attention for Object Tracking and Embedding Representation (SCOUTER), to extract relevant image features. These extracted features are then concatenated and passed through the Pairwise Matching module to compare the query image with the support image, enabling the classification of DR severity and attention maps to enhance interpretability by focusing on critical retinal regions that influence the classification.

Slot-based Cross-attention for Object Tracking and Embedding Representation (SCOUTER) effectively enhances model interpretability by generating visual explanations. This approach utilizes an attention mechanism to produce attention maps, visually highlighting the most influential image regions in the classification process. By segmenting images into slots, SCOUTER can focus on specific areas and generate attention maps that indicate the relevance of these regions for classification. This visual representation makes the model's reasoning more transparent, improving accuracy and interpretability. Figure 2 illustrates the MTUNet diagram.

In this study, we trained our model on the OCTA dataset using a more accurate pre-trained model selected from the previous phase. We fine-tuned the model's hyperparameters, including the number of attention slots, learning rate, number of epochs, and batch size. Using the PyTorch library, our experiments were conducted on a Google Colab Pro instance with 24GB GDDR6 memory.

Evaluation

To evaluate the performance of the method, various evaluation metrics, namely accuracy, sensitivity, specificity, and area under the curve (AUC) were employed, which are defined as follows. TP, TN, FP and FN are True Positive, True Negative, False Positive and False Negative, respectively.

Accuracy = (TP + TN)/(TP + TN + FN + FP)

Sensitivity = TP/(TP + FN)

Specificity = TN/(TN + FP)

## Results

The ICC between two specialists to make a ground truth was 0.91. Pre-processing was the first step in the analysis and the number of OCTA images after augmentation was doubled.

**Figure 3:**
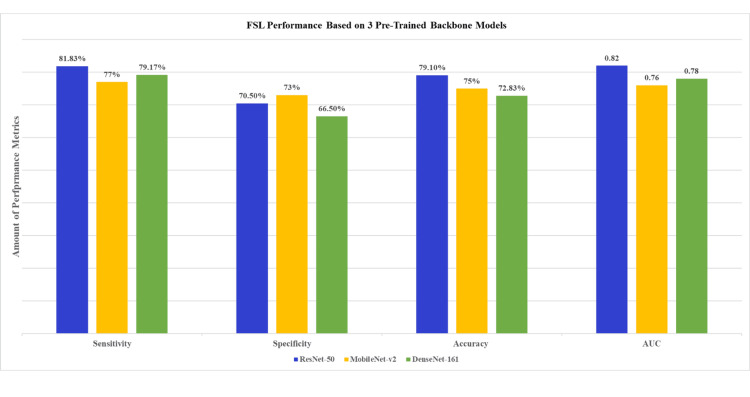
Sensitivity, specificity, accuracy and area under the curve (AUC) of Few-Shot Learning (FSL) prototyping models for ResNet-50, MobileNet-v2 and DenseNet-161 as pretained backbone Performance comparison of Few-Shot Learning (FSL) prototype models was conducted using three pre-trained backbone architectures: ResNet-50, MobileNet-v2, and DenseNet-161. The evaluated metrics included Sensitivity (ability to correctly identify Proliferative Diabetic Retinopathy (PDR)), Specificity (ability to correctly identify Non-Proliferative Diabetic Retinopathy (NPDR)), Accuracy (overall classification correctness), and Area Under the Curve (AUC) (a measure of the model's ability to distinguish between classes). ResNet-50 demonstrated the highest sensitivity and AUC.

**Figure 4:**
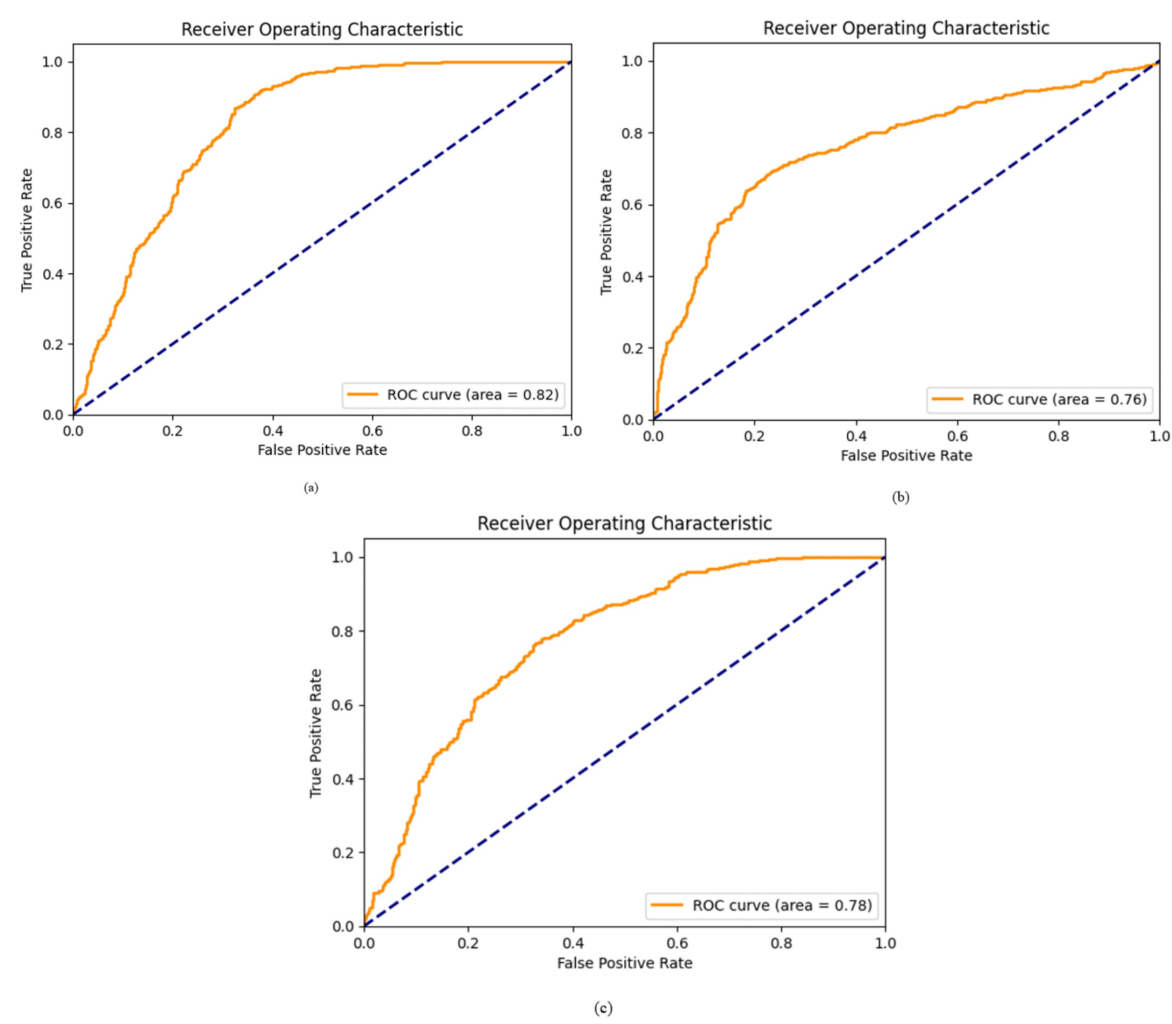
Comparison of pre-trained models' performance in diabetic retinopathy classification using few-shot learning (FSL) on Optical Coherence Tomography Angiography (OCTA) images. (a) ResNet-50, (b) MobileNet-v2, and (c) DenseNet-161 Receiver Operating Characteristic (ROC) curves illustrate the performance of pre-trained models for diabetic retinopathy (DR) classification on Optical Coherence Tomography Angiography (OCTA) images within a few-shot learning (FSL) framework. (a) ResNet-50 exhibits the highest Area Under the Curve (AUC = 0.82), signifying superior accuracy and robustness in distinguishing between DR classes. (b) MobileNet-v2 demonstrates a balanced performance with a respectable AUC of 0.76, making it suitable for resource-constrained environments where computational efficiency is paramount. (c) DenseNet-161 prioritizes high sensitivity for detecting Proliferative Diabetic Retinopathy (PDR), achieving an AUC of 0.78. This characteristic enhances the model's ability to identify critical cases, which is crucial for timely intervention and patient management.

Figure 3 shows the FSL prototyping performance test results for three pre-trained backbone models: ResNet-50, DenseNet-161, and MobileNet-v2. The sensitivity, specificity, accuracy, and AUC were 76.17%, 81.83%, 70.5%, and 0.82 for ResNet-50; 72.83%, 79.17%, 66.5%, and 0.78 for DenseNet-161; and 75%, 77%, 73%, and 0.76 for MobileNet-v2, respectively. Figure 4 shows each pre-trained model's Receiver Operating Characteristic (ROC) curve. Given the highest AUC, ResNet-50 was selected as the pre-trained model for MTUNet as the XAI component.

The MTUNet was fine-tuned by balancing resource utilization, parameter complexity, and performance by selecting seven slots, a learning rate of 0.001, 150 training epochs, and a batch size of 64.

**Figure 5:**
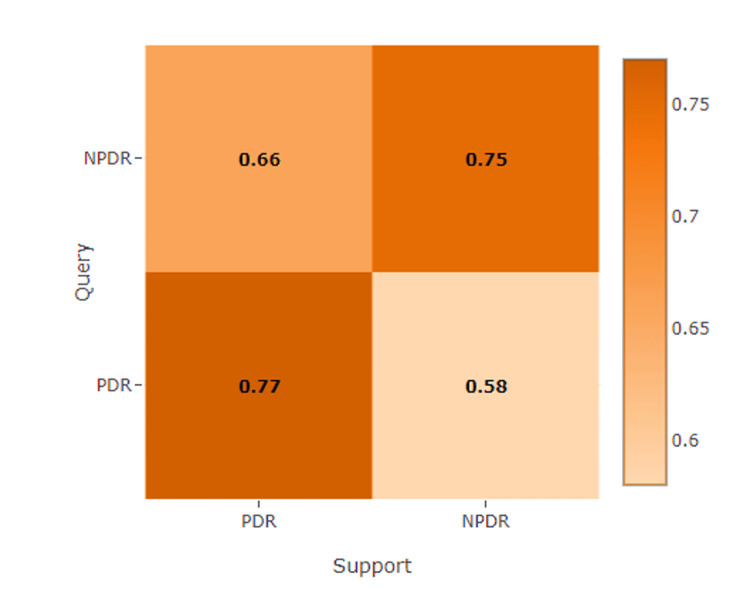
Extracted Matching point matrix from Match-Them-Up Network (MTUNet) for sample queries of Proliferative Diabetic Retinopathy (PDR) and Non-Proliferative Diabetic Retinopathy (NPDR) from the test set Similarity score matrix extracted from the Match-Them-Up Network (MTUNet) for Proliferative Diabetic Retinopathy (PDR) and Non-Proliferative Diabetic Retinopathy (NPDR) query and support samples from the test set. The diagonal elements represent the model's ability to match query images to their respective classes, while off-diagonal elements indicate the likelihood of misclassification. Higher scores along the diagonal (0.77 for PDR to PDR and 0.66 for NPDR to NPDR) reflect the model's effective feature extraction and matching capabilities.

Figure 5 shows the best matching scores between the supports of the PDR and NPDR classes and sample queries of the PDR and NPDR types from the test set query.

**Figure 6:**
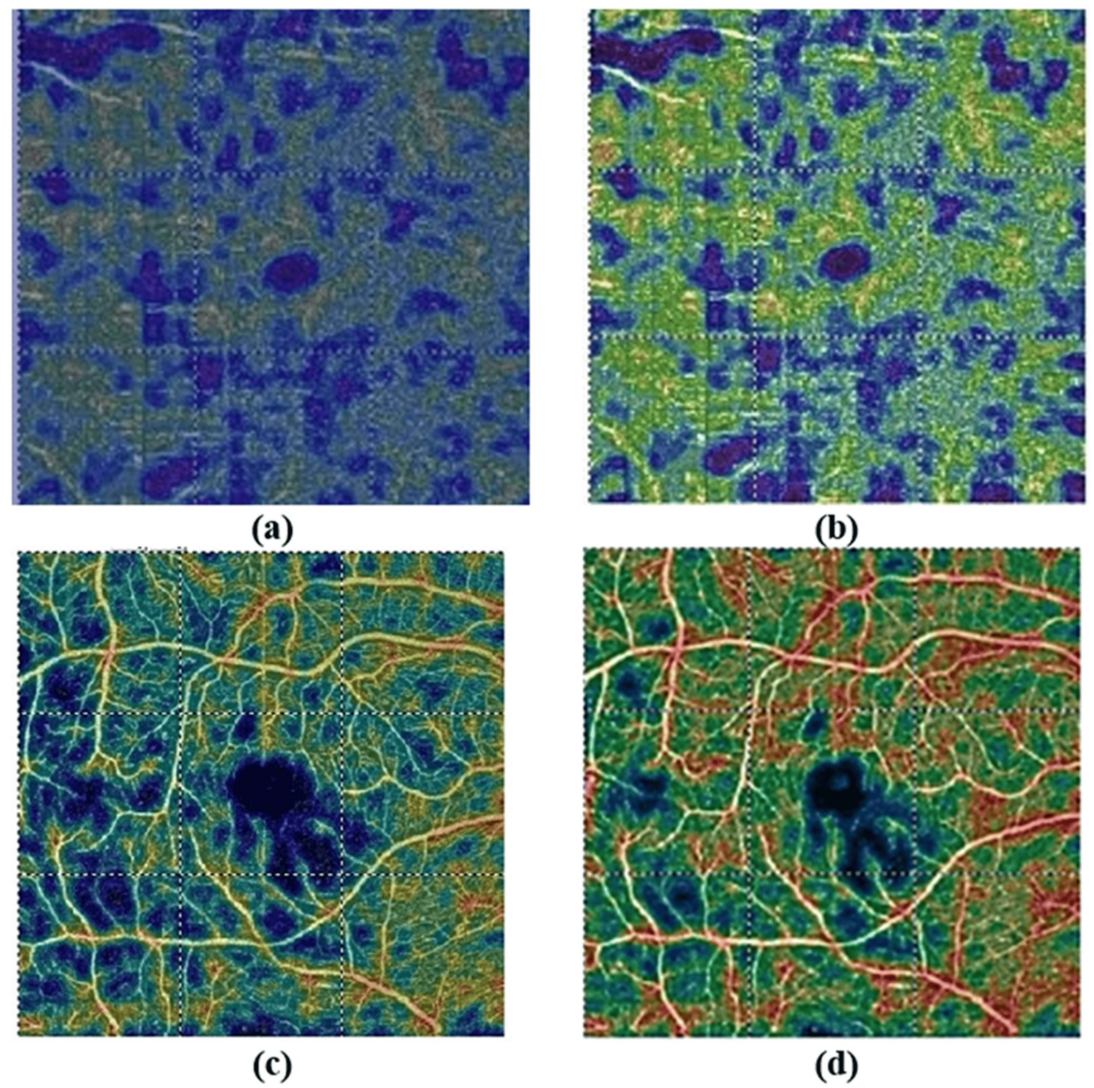
Enhanced Visualization of Non-Proliferative Diabetic Retinopathy (NPDR) and Proliferative Diabetic Retinopathy (PDR) Using Match-Them-Up Network (MTUNet): Highlighting Non-Perfusion and Neovascularization Regions Illustration of Non-Proliferative Diabetic Retinopathy (NPDR) and Proliferative Diabetic Retinopathy (PDR) images before and after Match-Them-Up Network (MTUNet) processing, highlighting attention regions. (a) Original NPDR image, (b) MTUNet-enhanced output for NPDR, emphasizing regions of non_perfusion area (red regions), (c) Original PDR image, and (d) MTUNet-enhanced output for PDR, with attention focused on neovascularization areas (red points)

Figure 6 illustrates the attention points in the PDR and NPDR. Figures 6A, 6C represent the original images, and Figures 6B, 6D show the attention points.

## Discussion

This study aimed to classify NPDR and PDR based on 206 OCTA images using a prototypical FSL algorithm. FSL can overcome some of the key challenges in deep learning because it depends on large datasets for the model to enhance generalization through limited labelled data. Three pre-trained models, ResNet-50, MobileNet-v2, and DenseNet-161, were evaluated to strike a balance between model complexity and performance in feature representation. ResNet-50 demonstrated superior performance by achieving higher accuracy and AUC compared to the other two models (Figure 3 and Figure 4).

To enhance interpretability, this study incorporated XAI through MTUNet, an intrinsic method that can explain the work of the model. The matching score between the support set and query for PDR and NPDR was 0.78 and 0.75, respectively, thus proving robust even against low-quality images (Figure 5). In the grading of NPDR, MTUNet focused on nonperfusion area (NPA), an important biomarker of ischemia in diabetic retinopathy. As DR progresses, capillary damage increases the degree of ischemia, further aggravating oxygen supply to the retina. Larger NPAs are associated with a higher risk of PDR progression. In the PDR classification, the model focused on neovascularization, characterized by the growth of new blood vessels in response to ischemia and is a well-known sign of the advanced stages of DR. The findings highlight that the model has provided essential features that facilitate early-stage classification of DR, which may inform and enhance clinical intervention strategies (Figure 6).

Alam et al. [11] classified control versus mild NPDR, reporting BVD, BVC, BVT, and FAZ areas as the most effective features. Our findings also demonstrate that features such as BVD, BVC, and neovascularization play a role in the staging of DR. This is particularly important in distinguishing between NPDR and PDR. Indeed, OCTA is important in providing quantitative insights and is a valuable tool for the early detection and staging of diabetic retinopathy [27,28].

Khalili et al. [12] used an SVM classifier optimized by a genetic evolutionary algorithm on OCTA vascular density maps, achieving 90% accuracy in discriminating between PDR and NPDR, where FAZ, BVD, BVT, neovascularization, and BVC were considered essential features. However, the structural characteristics of blood vessels were also identified as an essential feature of the machine learning-based method, denoting a comprehensive focus on the functional and structural aspects of the retinal vasculature in DR progression detection. Both studies focused on quantitative measurements of vascular density and changes in retinal blood vessels. Although their accuracy was higher than our results, their approach required manual segmentation, which can be time-consuming and prone to error. By contrast, the deep learning approach automatically extracts features, potentially offering a more efficient and scalable solution.

Transfer learning with VGG16 architecture for OCTA-based DR detection was applied by Ryu et al. [29]. Their external validation results showed 70.83 ± 0.021% accuracy, 67.23 ± 0.026% sensitivity, and 73.96 ± 0.026% specificity. Zang et al. [15] developed DcardNet, a densely connected neural network for classifying no DR, mild and moderate NPDR, severe NPDR, and PDR. The sensitivity, specificity, and overall classification accuracy were 95.7, 85.0, and 71.0%, respectively. Although the results of these studies are better than ours, their approach requires a larger dataset and more complex network architecture.

XAI is an effort to provide insight into deep neural networks, determine which part of an image is essential to make a decision and change the black box to a white box. Sandhu et al. [30] used Class Activation Maps (CAM) to visualize regions influencing the network's decisions in OCTA-based DR classification. These regions contain areas of capillary non-perfusion or ischemia, which reflect damage to the retina, microaneurysms, hemorrhages, or other vascular abnormalities that are classic in DR. In advanced stages, such as PDR, Grad-CAM highlights regions where neovascularization occurs, indicating abnormal new blood vessel growth, a key marker of disease severity. This targeted focus further increases the interpretability of the model because it allows clinicians to visually see which parts of the retinal image contribute the most to DR classification.

In contrast, our research utilized ResNet-50 with attention mechanisms and the MTUNet model, along with SCOUTER, to pay special attention to critical retinal areas, such as the non-perfusion area during NPDR detection and neovascularization regions in the identification of PDR, working toward aligning with the crucial features of the retina, including ischemia and abnormal blood vessel architecture.

To our knowledge, FSL has not been previously applied for DR stage prediction, and this study demonstrates its significant potential for diagnosing and staging DR. The need for fewer labelled samples could speed up AI-assisted diagnosis system development in clinical settings to obtain large, annotated datasets is challenging. Additionally, the explainability offered by MTUNet could increase clinicians' trust in the model's decisions, potentially leading to broader acceptance of AI tools in ophthalmology.

However, the current study has some limitations. The employed dataset was acquired from one device, which may have introduced design biases. External validation must be performed at multiple centers to evaluate the results. In addition, our OCTA images had a low resolution; therefore, better results could be obtained using higher-quality images. Finally, one can optimize the hyperparameters for MTUNet by using more powerful computational resources.

## Conclusions

This study highlighted the potential of integrating FSL with XAI to significantly advance DR diagnostics using OCTA images. The developed framework demonstrated robust classification performance, effectively distinguishing between NPDR and PDR stages, even with limited training data. The system achieved reliable and interpretable results by leveraging the ResNet-50 model within the MTUNet architecture, identifying key retinal biomarkers such as non-perfusion areas and neovascularization. These insights facilitate timely and accurate classification of DR stages, potentially reducing delays in diagnosis and the initiation of appropriate treatment. This advances diagnostic workflows, improves disease staging, supports personalized treatment plans because of its effectiveness with limited data, and enhances accessibility in resource-constrained settings.
